# Interdisciplinary recommendations for recurrent hyperkalaemia: insights from the GUARDIAN-HK European Steering Committee

**DOI:** 10.1093/ehjcvp/pvaf055

**Published:** 2025-07-21

**Authors:** Gianluigi Savarese, María Jesús Izquierdo, Clara Bonanad, Aaron Wong, Roland Schmitt, Pietro Manuel Ferraro, Francesco Dentali, James O Burton, Giuseppe Rosano

**Affiliations:** Department of Clinical Science and Education, Södersjukhuset; Karolinska Institutet, Sjukhusbacken 10, 11883 Stockholm Sweden; Department of Nephrology, Hospital Universitario de Burgos, Burgos 09006, Spain; Department of Cardiology, Hospital Clínico Universitario de Valencia, Valencia 46010, Spain; Instituto de Investigacion Sanitaria INCLIVA, Valencia 46010, Spain; Department of Cardiology, Princess of Wales Hospital, Bridgend CF31 1RQ, UK; Department of Nephrology and Hypertension, University Hospital Schleswig-Holstein, Kiel 24105, Germany; Department of Medicine, Section of Nephrology, Università degli Studi di Verona, Verona 37126, Italy; Division of Internal Medicine, Medical Center, Ospedale di Circolo & Fondazione Macchi, ASST Sette Laghi 21100, Varese, Italy; Department of Cardiovascular Sciences, University of Leicester, Leicester LE1 7RH, UK; Department of Human Sciences and Promotion of Quality of Life, Chair of Pharmacology, San Raffaele University of Rome, Rome 00166, Italy; Center of Clinical and Experimental Medicine, IRCCS San Raffaele, Roma 20132, Italy

**Keywords:** Hyperkalaemia, Renin–angiotensin system, Chronic kidney disease, Cardiovascular disease

## Abstract

Recurrent hyperkalaemia (HK) is associated with increased morbidity and mortality, and is common among patients with cardiorenal disease. Many of these patients require renin–angiotensin–aldosterone system inhibitor (RAASi) therapies that further enhance the risk of HK. Every acute HK episode constitutes an opportunity to treat and prevent recurrent HK. This report aims to support multidisciplinary team efforts in managing patients who may be affected by recurrent HK. A panel of nine European experts in the management of HK (four nephrologists, four cardiologists, one internist) reviewed existing guidance and evidence on the diagnosis and management of HK at a face-to-face (26th September 2023) and two virtual meetings (24th January and 14th March 2024). The panel developed 10 consensus recommendations and a management algorithm across three domains: duty of care, identifying patients at risk of HK recurrence and managing the risk of HK recurrence. Early identification and management of those at risk of recurrent HK will improve clinical outcomes but requires an interdisciplinary, co-ordinated approach. Disease-modifying therapies such as RAASi should no longer be considered reversible causes of HK, and efforts should be taken to up-titrate these to guideline-directed target doses even in the setting of an acute HK event. Every acute HK episode constitutes an opportunity to treat and prevent recurrent HK, contributing to long-term clinical benefits. The recommendations, intentionally broad in scope, complement existing management guidelines and plans, fostering a collective responsibility among healthcare professionals managing patients with HK.

## Introduction

Hyperkalaemia (HK) has acute life-threatening consequences; if left unmanaged, it can result in weakness, paralysis, arrhythmias or even cardiac arrest. Long-term, HK is also associated with end-stage kidney disease (ESKD) and cardiovascular (CV) events including hospitalisations for heart failure (HF), ischaemic heart disease and stroke.^1,2^ HK is more prevalent in patients with chronic conditions, such as chronic kidney disease (CKD), diabetes, HF and hypertension compared with the general population, and many will experience recurrent HK after their first episode.^3^

Following an HK event, serum potassium levels of ≥5.0 mEq/L have been associated with increased risk of all-cause mortality and CV events compared with matched controls.^2^ All-cause mortality increases significantly for every 0.1 mEq/L rise in serum potassium ≥5.0 mEq/L,^1^ and severe HK (serum potassium ≥6.5 mEq/L) necessitating hospitalisation is associated with higher inpatient mortality rates (up to 31%).^4^ Acute hospitalisation and death are more frequent among patients with CKD (3.8- and 4.9-fold, respectively) or HF (2.8- and 3.4-fold, respectively) who experience an HK event compared with matched patients without HK.^5,6^

Patients with chronic cardiovascular or renal conditions are often treated with renin–angiotensin–aldosterone system inhibitor (RAASi) therapies that can elevate potassium levels, including angiotensin-converting enzyme inhibitors (ACEi), angiotensin receptor blockers (ARBs), angiotensin receptor-neprilysin inhibitors, and steroidal and non-steroidal mineralocorticoid receptor antagonists (MRAs). Nonetheless, because of their important clinical benefits in reducing disease progression, mortality, and morbidity, guidelines advocate the use of the maximum tolerated target doses of these medications and recommend managing HK through other measures (such as diet, diuretics, or potassium binders) rather than decreasing or stopping RAASi. Yet, withdrawal or down-titration of RAASi is common practice in HK management despite being associated with increased mortality, hospitalisations for HF or progression to ESKD.^7–9^ Effective HK management is, therefore, an absolute priority for these populations.^10–12^

Patients at risk of HK often receive fragmented healthcare because they typically have multiple long-term conditions and are managed by different specialty teams. The complexity of delivering integrated, co-ordinated care across multidisciplinary settings is associated with increased healthcare costs, adverse outcomes due to comorbidities (such as coronary or HF events, infections or poor glycaemic control in diabetes), and use of hospital services, including emergency department attendances.^13^ Currently, there is a lack of integrated management plans for patients presenting with HK,^14^ and treatment guidelines and recommendations on the prevention of HK are limited, often resulting in de-escalations or discontinuations of life-saving therapies, such as RAASi, despite clear guidance to the contrary from international specialist societies.^11,15–18^

We hereby provide our consensus recommendations, a management algorithm, and quality indicators (QIs) to enhance the identification and management of patients with an elevated clinical risk of recurrent HK. These recommendations, intentionally broad in scope, aim to complement existing management guidelines and plans, fostering a collective responsibility among healthcare professionals (HCPs).

## Methods

**Figure 1:**
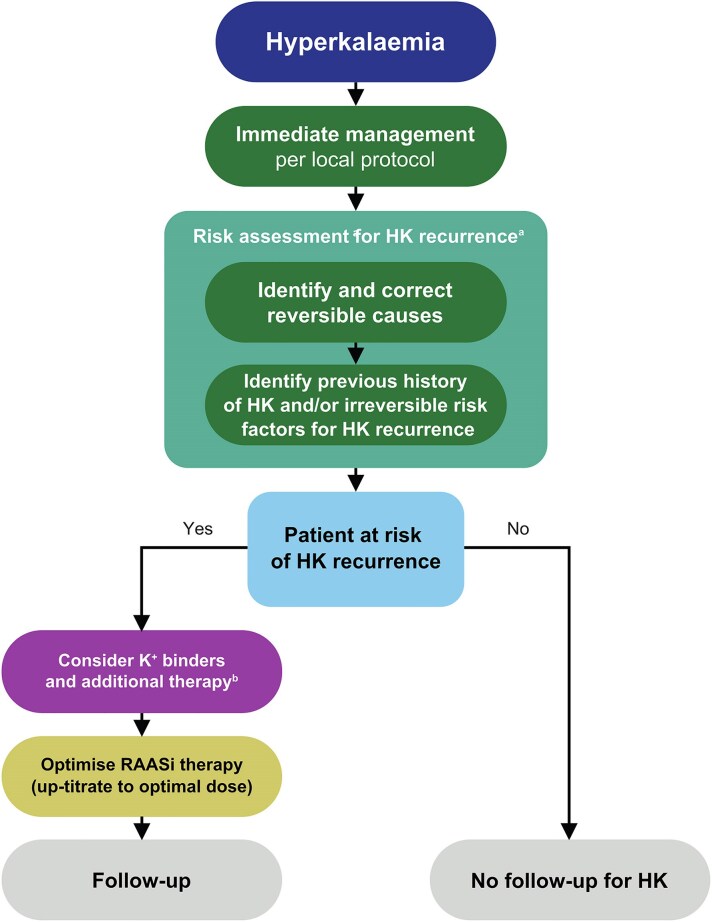
Management algorithm for patients presenting with HK. ^a^Risk factors for HK recurrence include long-term conditions such as chronic kidney disease, cardiovascular disease and diabetes. Long-term therapies for chronic conditions should be considered qirreversible factors even if they increase the risk of HK (such as non-steroidal anti-inflammatory drugs for osteoarthritis or RAASi therapies for heart failure). ^b^Consider appropriate treatment according to the underlying pathology and preferences of the patient. HK, hyperkalaemia; RAASi, renin–angiotensin–aldosterone system inhibitor.

Our panel of nine European experts in the management of HK (four nephrologists, four cardiologists, one internist) used our collective experiences and undertook a comprehensive review of existing guidance and evidence on the diagnosis and management of HK at a face-to-face (26th September 2023) and two subsequent virtual meetings (24th January and 14th March 2024). We then developed recommendations and supporting QIs across three domains in relation to recurrent HK: duty of care, identifying patients at risk of HK recurrence, and managing the risk of HK recurrence (*Table 1*). Based on the recommendations, we also provide a simplified management algorithm for patients presenting with hyperkalaemia (*Figure 1*). Although several definitions for HK exist,^15^ we defined recurrent HK as more than one episode of serum potassium >5.0 mEq/L or above the upper limit of normal range (per local laboratory values) per year,^10,19^ acknowledging that HK-associated adverse outcomes can manifest even at potassium levels of 5.1–5.4 mEq/L in patients with cardiorenal disease.^14^ The development of the QIs followed established methodologies.^20^

**Table 1:** Steering committee recommendations for the management of recurrent HK and QIs

**Domain 1: Duty of care**
Recommendation 1: Reducing the risk of recurrence should be standard practice in the management of HK, regardless of the setting
QI: Management and prevention of HK recurrence are included in HK management protocols
Numerator: Availability of HK management protocols that include a risk assessment for HK recurrence
QI: Proportion of patients treated for HK who have had their risk of HK recurrence evaluated
Numerator: Number of patients treated for HK who have had their risk of HK recurrence evaluated
Denominator: Number of patients treated for HK
Recommendation 2: It is the responsibility of all HCPs to evaluate and address the risk of recurrence of HK at every clinical encounter
QI: Proportion of HCPs managing patients with HK who have appropriate training in the management of HK recurrence
Numerator: Number of HCPs managing patients with HK who have appropriate training in the management of HK recurrence
Denominator: Number of HCPs who manage patients with HK
Recommendation 3: Every patient encounter should be an opportunity to optimize RAASi therapy, even in the context of an HK event
QI: Proportion of patients who are assessed for guideline-directed implementation of RAASi therapy
Numerator: Number of patients who are assessed for guideline-directed implementation of RAASi therapy
Denominator: Number of patients on RAASi
QI: Proportion of patients not on guideline-directed target dose of RAASi therapy with a documented reason
Numerator: Number of patients not on guideline-directed target dose of RAASi therapy with a documented reason for not being on GDMT
Denominator: Number of patients not on guideline-directed target dose of RAASi
**Domain 2: Identifying patients at risk of HK recurrence**
Recommendation 4: Long-term conditions, such as kidney disease, CVD and T2D, and an associated reliance on disease-modifying therapy that interferes with potassium homeostasis should be considered irreversible factors associated with HK
QI: Proportion of patients in whom the presence of risk factors for HK recurrence is documented
Numerator: Number of patients with HK in whom the presence of risk factors for HK recurrence is documented
Denominator: Number of patients with HK
QI: Educational programmes on the management of HK include discussion on irreversible factors and the importance of continuing disease-modifying therapy
Numerator: Availability of training on irreversible factors of HK recurrence and the importance of continuing disease-modifying therapy
Recommendation 5: In patients at risk of HK, a risk stratification tool for HK recurrence is required and could guide management
No additional QI required (see Recommendation 1)
**Domain 3: Managing the risk of HK recurrence**
Recommendation 6: The initial aim of management of HK should be to normalize serum potassium levels as needed and correct reversible precipitating factors
QI: Proportion of patients treated for HK who receive advice on reversible precipitating factors for HK
Numerator: Number of patients treated for HK who receive advice on reversible precipitating factors for HK
Denominator: Number of patients treated for HK
Recommendation 7: If there is no reversible factor, treatment to prevent recurrence of HK should be initiated
QI: Proportion of patients with HK who receive treatment according to local protocol for prevention of HK recurrence
Numerator: Number of patients with HK who receive treatment according to local protocol for prevention of HK recurrence
Denominator: Number of patients with HK
Recommendation 8: The risk of recurrence of HK should be reduced without discontinuing or down-titrating disease-modifying therapy such as RAASi
QI: Proportion of patients receiving individually optimized RAASi therapy in the presence of controlled potassium levels at discharge
Numerator: Number of patients receiving individually optimized RAASi therapy in the presence of controlled potassium levels at discharge
Denominator: Number of patients with HK and an indication for RAASi
QI: Proportion of patients treated for HK, who are not on target dose but with a clear post-discharge plan for GDMT
Numerator: Number of patients treated for HK with clear post-discharge plan for GDMT
Denominator: Number of patients treated for HK, who are not on target dose of GDMT
QI: Time from potassium normalisation to maximal tolerated guideline-directed target dose of RAASi therapy
Recommendation 9: Unless the cause of acute HK can be reversed, treatment for recurrent HK is likely to be indefinite
QI: Proportion of patients treated for HK who receive a potassium binder at follow-up while still at risk of HK recurrence (e.g. on RAASi therapy)
Numerator: Number of patients treated for HK and still at risk of HK recurrence who are on a potassium binder at follow-up
Denominator: Number of patients treated for HK and still at risk of HK recurrence seen at follow-up
Recommendation 10: Following normalisation using a potassium binder, additional monitoring of serum potassium levels beyond that for routine comorbidities should not be necessary
No specific QI recommended

CVD, cardiovascular disease; GDMT, guideline-directed medical therapy; HCP, healthcare professional; HK, hyperkalaemia; QI, quality indicator; RAASi, renin–angiotensin–aldosterone system inhibitor; T2D, Type 2 diabetes.

## Recommendations

### Duty of care

#### Recommendation 1. Reducing the risk of recurrence should be standard practice in the management of HK, regardless of the setting

HK poses an immediate threat to patient health and elevates the risk of subsequent HK, contributing to increased hospital admissions and mortality. One in five patients with CKD, HF, resistant hypertension or diabetes will have recurrent HK, with increasingly shorter times between events,^3^ and this increases the risk of RAASi therapy down-titration or discontinuation.^7^ Reducing the risk of HK recurrence could translate into a diminished burden of acute events and optimal utilisation of cardiorenal-protective drugs. From a healthcare system perspective, one can expect tangible cost reductions associated with the acute management of recurrent HK, hospitalisations and the potential chronic complications that may arise.

Despite recognition that preventing further HK events is crucial, local hospital protocols for the emergency management of acute HK do not always incorporate guidance on prevention of further events. Several national and international guidelines propose measures to prevent HK or its recurrence (see Supplementary material online, *Table S1*).^10,11,15-19,21–25^ They advocate for evaluating the need for therapies that can cause HK and monitoring serum potassium before and after initiating such therapies, while also aiming for a healthy low-potassium diet in at-risk populations, e.g. patients with advanced CKD, and educating patients about HK. Therapies that can be considered to reduce the risk of HK include sodium bicarbonate in patients with CKD, potassium-wasting diuretics, and potassium binders. However, these recommendations either focus on specific subgroups, such as those on RAASi^10,17,21–23^ or do not have the same stringency as for the initial management of acute HK.^10,11,15-17,19,21–25^ During an acute HK event, reversible causes, such as acute kidney disease or metabolic acidosis, can be identified, and the likelihood of a subsequent event can be evaluated. There is an opportunity for all HCPs treating patients with HK to implement proactive measures to prevent further events and subsequent hospitalisations, and enable collaborative discussions on a tailored plan for ongoing care. Two QIs are proposed to support this recommendation: the incorporation of management and prevention of recurrence into HK management protocols, and the proportion of patients treated with HK who have had their risk of HK recurrence evaluated (*Table 1*).

#### Recommendation 2. It is the responsibility of all HCPs to evaluate and address the risk of HK recurrence at every clinical encounter

HCPs equipped with the necessary training should assume collective accountability for HK management, acknowledging the diversity in practice settings. While primary care practitioners (PCPs) and specialists, such as nephrologists, endocrinologists and cardiologists, may play pivotal roles, it is crucial to empower other members of the multiprofessional team, such as pharmacists, dieticians and nurses, to actively participate by reviewing risk factors for HK, monitoring and facilitating appropriate referrals when necessary. Effective communication and collaboration among PCPs, patients and relevant specialists, emphasising the risks of HK recurrence and the importance of continuous treatment, are essential. Combined nephrology and cardiology clinics for patients with CKD and HF show that integrated approaches ensure cohesive responsibility across all HCPs, preventing conflicting information and promoting optimal patient care. These clinics enable a collaborative approach to manage multiple cardiorenal issues and can increase adherence to guideline-directed medical therapy (GDMT) with no associated clinically significant deterioration in kidney function and HK.^26^ A suitable QI for this recommendation is to ensure HCPs have access to appropriate training on managing patients at risk of HK (*Table 1*).

#### Recommendation 3. Every patient encounter should be an opportunity to optimize RAASi therapy, even in the context of an HK event

To ensure patients remain on life-saving GDMTs, it is essential to view every patient encounter as an opportunity to optimize disease management. The STRONG-HF randomized trial showed that patients hospitalized for HF who were previously intolerant to maximum doses of these therapies can still achieve target dosage of GDMTs (RAASi and/or beta blockers) after high-intensity optimisation.^27^ An additional 8%–9% more patients could have achieved optimal dosing of RAASi if not for HK, and the authors considered that administration of the newer potassium binders, which were not available in most of the cases at the time of the STRONG-HF trial, could be appropriate for these patients.^27^ Recent evidence suggests that, with the use of potassium binders, RAASi can be maintained or up-titrated following an HK event (see Supplementary material online, *Table S2*),^8,28-31^ and this should be covered in HK management protocols. Departments can, therefore, evaluate their performance against this recommendation by identifying the proportion of patients who have their RAASi dose assessed and, if not on target dose, have a documented reason for suboptimal therapy (*Table 1*).

### Identifying patients at risk of HK recurrence

#### Recommendation 4. Long-term conditions, such as CKD, cardiovascular disease, and Type 2 diabetes, and an associated reliance on disease-modifying therapy that interferes with potassium homeostasis should be considered non-reversible causes of HK

There are several causes of acute HK, mostly reversible and frequently co-existing (see Supplementary material online, *Table S3*). After excluding pseudohyperkalaemia, which is probably the most common reason for apparent HK, causes can be broadly divided as related to decreased potassium excretion, transcellular shift of potassium (from the intracellular to the extracellular compartment) and increased potassium intake.^32–35^ These causes can be further classified as reversible or non-reversible factors, depending on whether they are transient or not. Note that although some conditions, such as Addison’s disease, can be treated, which will reduce the risk of HK, the underlying pathology persists and, therefore, these can be considered as non-reversible factors. Many of the reversible factors, such as diet or certain medications, may be of relevance in adult patients with CKD and are unlikely to cause HK in those with normal kidney function.^32,35^

The most common risk factors for recurrent HK are non-reversible. These include chronic conditions such as HF, advanced CKD (stage G3b and above), T2D and hypertension.^36,37^ These risk factors have been found to be independent of the use of drugs that further elevate HK risk, such as MRAs, non-steroidal anti-inflammatory drugs, ACEi, and ARBs.^36,37^ A previous history of HK and its severity also act as independent risk factors for subsequent events.^38^ These risk factors are additive in determining the risk of HK for an individual patient. Owing to the need to balance the management of HK with that of other severe, chronic comorbidities, there is a requirement to explore alternative interventions before reducing disease-modifying drugs that are essential for optimal patient care. Down-titration or discontinuation of RAASi therapies should be discouraged, as these drugs improve outcomes in HF and proteinuric kidney disease. Alongside other conservative measures, potassium binders, sodium–glucose co-transporter 2 inhibitors and neprilysin inhibitors can aid in maintaining the use of RAASi therapy.^39,40^ Suitable QIs for this recommendation include the proportion of patients in whom the presence (or absence) of risk factors for HK recurrence is documented, and the availability of education or training on irreversible factors for HK recurrence and the importance of continuing disease-modifying therapy (*Table 1*).

#### Recommendation 5. In patients at risk of HK, a risk stratification tool for HK recurrence is required and could guide management

An appropriate tool to predict the risk of developing HK could increase the likelihood of timely identification, appropriate management of HK, and effective planning to mitigate risk. There are several well-calibrated tools available (see Supplementary material online, *Table S4*). These tools, however, are limited to specific subgroups, and require further external validation and clear, practical guidance for potassium monitoring requirements in a range of clinical settings.

Chronic HK is poorly defined with no consensus on the degree, duration and frequency of elevated potassium values that define chronicity.^19^ Nonetheless, a tool to identify patients at risk of HK recurrence may be feasible with the availability of recent data on the risk factors for recurrent HK. Given that 20% of patients will have recurrence,^3^ this tool would facilitate patient identification, enabling HCPs to formulate an effective risk mitigation plan, initiate treatment without waiting for a second HK event and manage potassium levels without interrupting life-saving therapies, such as RAASi.

### Managing the risk of HK recurrence

#### Recommendation 6. The initial aim of management of HK should be to normalize serum potassium levels as needed and correct reversible precipitating factors

Initial HK management aims to stabilize the patient and normalize potassium levels to prevent arrythmias and death. Acute management includes conventional treatments (calcium gluconate or calcium chloride, sodium bicarbonate, insulin/dextrose, salbutamol and/or diuretics), novel potassium binders [patiromer or sodium zirconium cyclosilicate (SZC)] where available for this specific indication, and dialysis for severe, refractory cases.^14,41^ Correcting reversible factors, such as diet, constipation, metabolic acidosis and unnecessary excessive medications, should be a key initial focus in the management of HK. To reduce the risk of HK recurrence, patients should receive appropriate advice on these factors.^16^ The proposed QI includes the proportion of patients who have received advice on risk factors for HK (*Table 1*).

#### Recommendation 7. If there is no acute reversible factor, treatment to prevent recurrence of HK should be initiated

Patients with HK and non-reversible risk factors are particularly susceptible to HK recurrence (43% of patients with CKD or HF had HK recurrence within 6–8 months^5,6^) and require treatment to reduce the risk of a further event. Currently, treatment options to manage recurrent HK include reducing potassium intake through diet, increasing excretion with potassium binders and diuretics, increasing intracellular shift and reducing acidosis with oral sodium bicarbonate, and changing dialysis regimens in eligible patients. A low-potassium diet may conflict with a heart-healthy diet,^19,42^ and the practice of restricting potassium-rich diets is being re-evaluated, with proposals for a more nuanced dietary approach focusing on reducing intake of non-plant sources of potassium.^39^ The recently developed oral potassium binders, SZC and patiromer, have demonstrated efficacy in maintaining normokalaemia for at least a year^43,44^ and have been found to be cost-effective treatments for managing persistent HK.^45,46^ However, large post-market authorisation studies of these binders are lacking. Potassium-wasting diuretics are particularly useful when diuresis or control of hypertension is desired, though their use otherwise is limited by kidney function.^19^ The use of sodium bicarbonate is disputed because of a lack of evidence, the risk of sodium overload and an indication restricted to patients with metabolic acidosis/low serum bicarbonate.^16^ Finally, in the dialysis population, changing dialysis frequency and regimen may be an option in managing pre-dialysis potassium levels,^47^ but these changes are limited by healthcare resources availability and by worsening patient outcomes when low-potassium dialysate (1.0–1.5 mmol/L) is used.^19^ Current guidelines on the prevention of HK are not uniform or individualized enough, and do not cover a wide range of patient populations/scenarios (see Supplementary material online, *Table S1*), reflecting the complexity of the issue and the lack of robust evidence. Local protocols should therefore reflect local and national practices, and departments should evaluate how many patients receive appropriate treatment to prevent HK recurrence (*Table 1*).

#### Recommendation 8. The risk of recurrence of HK should be reduced without discontinuing or down-titrating disease-modifying therapies such as RAASi

Down-titration/discontinuation of RAASi should not be considered as the preferred or default management option for HK. Current guidelines (see Supplementary material online, *Table S5*) prioritize continuation of RAASi while managing the risk of HK by lowering serum potassium through alternative strategies. These include regular monitoring of serum potassium, avoiding the combination of ACEi and ARBs, treating underlying conditions that lead to HK, and considering the initiation of an approved potassium-lowering agent.^17,18,21,23,24,48^ The underlying reason is the higher mortality rates and/or worse composite cardiorenal outcomes associated with RAASi down-titration or discontinuation after an HK event.^7–9,49^ Prior recommendations to stop or down-titrate RAASi, even temporarily, were made before the emergence of the newer potassium binders SZC and patiromer. Evidence from multiple studies supports the use of potassium binders to reduce HK recurrence while keeping patients on RAASi (see Supplementary material online, *Table S2*).^28–30,50–53^ Although down-titration may be necessary in the acute setting, it should not be a long-term strategy and therapies should be promptly reinstated to the maximum tolerated target doses. Appropriate QIs for this recommendation include the proportion of patients who, after an HK event, are discharged with optimized RAASi therapy or with a clear plan for GDMT, if not on target dose. Time from the HK event to maximal tolerated target therapy is also an important QI (*Table 1*).

#### Recommendation 9. Unless the cause of acute HK can be reversed, treatment for HK is likely to be indefinite

While some causes of HK are reversible and treatment could be discontinued, patients with chronic conditions, such as CKD and HF, face a risk of recurrence. In such cases, maintenance of potassium-lowering therapies might be required indefinitely, with assessment of their efficacy (including adherence) over time. Currently, there is insufficient evidence to advocate for specific recommendations regarding the optimal duration of treatment. Departments should, however, track the continuation of potassium binders to ensure patients at high risk of HK recurrence receive ongoing treatment in line with current guidelines (*Table 1*, Supplementary material online, *Table S1*).^11,15,16,24^

#### Recommendation 10. Following normalisation using a potassium binder, additional monitoring of serum potassium levels beyond that for routine comorbidities should not be necessary

All patients should undergo monitoring aligned with guidelines and tailored to their underlying conditions. Guidelines recommend frequent potassium monitoring for patients at risk of HK and during initiation/titration of RAASi, such as 2–4 times a year for patients with CKD, HF and/or diabetes, and within 1–4 weeks after RAASi initiation (see Supplementary material online, *Table S6*).^10,11,15–19,21,23–25,41,48^ However, real-world monitoring often falls short of goal-directed management owing to a variety of factors, such as insufficient patient education, limited healthcare access, inconsistent recommendations or a lack of access to relevant guidelines.^54–57^ Following an HK event, patients not started on long-term potassium management therapy are likely to need an increased frequency of monitoring to ensure any reversible factor has been addressed. For patients in whom the risk of recurrence has been addressed with adequate potassium-lowering treatment, such as potassium binders, a regular monitoring regimen can be followed until the efficacy, tolerance, adherence and safety of therapy have been evaluated. After the HK has been resolved, patients can resume guideline-directed monitoring appropriate for their underlying disorder, unless there are changes in therapy, diet or other patient factors, such as progression of CKD. If RAASi is reintroduced, changed or withdrawn during an HK event, prompt follow-up with potassium measurement, preferably by the initial prescribing specialist team, is recommended per the guidelines. Potassium-monitoring behaviours specifically for HK are intrinsically challenging to audit because serum potassium is part of kidney function evaluation, frequent among patients with multiple comorbidities, hence there are no specific QIs for this recommendation.

## Discussion

Prevention of future HK is only briefly discussed in the management of acute HK, if at all, with the focus being on identifying reversible causes.^16,25^ However, the prevention of recurrent HK is receiving more attention among specific specialties, primarily cardiology and nephrology, because of the association between HK and discontinuation/down-titration of GDMTs, such as RAASi. There is a consequent drive to ensure patients are not deprived of these life-saving disease-modifying therapies while managing HK; this is especially important now that effective potassium-lowering therapies are available. Because of the disconnect between pathways to manage acute HK and those of chronic or recurrent HK, there is a risk of missing a clear opportunity at the first HK event to prevent further episodes, while maintaining appropriate GDMT for other chronic conditions. Our recommendations are, therefore, purposefully broad in scope, covering the whole patient journey to ensure they are relevant to any HCP managing patients at risk of or with HK. It is advisable for HCPs to evaluate how these recommendations can be incorporated into their own clinical practice in a pragmatic but impactful way. We provide a simplified management algorithm that can be used as a basis for discussions with relevant stakeholders and that can be built upon as new evidence emerges (*Figure 1*).

Patients with HK should be managed according to local protocols with the aim of avoiding any acute HK-related adverse events (Recommendation 6). Such protocols should be aligned with current guidance on RAASi therapy, i.e. trying other measures to reduce potassium levels before stopping or down-titrating RAASi therapy (Recommendation 8). As soon as the patient is deemed stable and any acute treatment, if applicable, has been completed, the risk of HK recurrence should be formally assessed (Recommendations 1, 2, 4, and 5). Most patients presenting with HK are likely to have risk factors for recurrence and should be considered for long-term potassium-lowering treatment (Recommendation 7). Local guidelines and prescribing practices, as well as individual patient factors and preferences, will dictate the most appropriate treatment. In accordance with current guidelines, a review to optimize RAASi therapy should follow, not least to ensure any cessation or down-titration during the management of acute HK is reversed appropriately (Recommendations 3 and 8). Providing multidisciplinary care may be of great benefit at this stage to co-ordinate treatment across disease areas and ensure patients are on GDMT.^26^ The treating HCP should consider whether the need for ongoing potassium-lowering treatment can be assessed in a routine appointment (Recommendations 9 and 10) or a sooner appointment is required owing to any change in RAASi therapy.

Optimal implementation of our recommendations in clinical practice should start by identifying areas for improvement and appropriate solutions using a baseline assessment against the proposed QIs. Early engagement with stakeholders will ensure the suggested solutions are realistic and achievable within the resourcing and financial constraints of individual healthcare systems. Following any changes in processes or infrastructure, regular re-evaluation of practice against the QIs is also recommended.

Further research on the management of HK could focus on robust tools to predict the risk of recurrence and provide more granular monitoring recommendations. This would empower HCPs to manage patients more effectively, especially when maintaining RAASi therapy is required. Furthermore, dedicated multidisciplinary teams for the management of recurrent HK may benefit patients further by ensuring treatment is patient-centred. Results from studies investigating the effectiveness of multidisciplinary clinics in managing patients with CV, renal and/or metabolic comorbidities are encouraging,^26,58–60^ and major associations such as the American Heart Association^61^ and Kidney Disease: Improving Global Outcomes^11,48^ have identified that multidisciplinary care is key to improving patient outcomes. In addition, a comparison of the relative benefits and indications for the various long-term potassium-lowering treatments is also warranted. Potassium binders show promising results in allowing the continuation of cardiorenal therapies, but further research is needed to confirm this benefit and evaluate whether there is associated reduction of long-term morbidity. Similarly, the pleiotropic effects of sodium–glucose co-transporter 2 inhibitors and other CKD/HF therapies are still being explored. It is unclear how their use will affect the prevention and management of HK in the future.

## Conclusions

There is a need for a paradigm shift in the management of HK to improve patient outcomes and optimize healthcare resources. Traditionally, emphasis has been on acute interventions to lower potassium levels during HK events, and life-saving therapies that can impair potassium elimination are frequently discontinued in patients who need them the most. Adopting a comprehensive and preventive approach during an acute HK event will not only mitigate immediate risks but also contribute to long-term clinical benefits.

## Supplementary Material

pvaf055_Supplementary_Data

## Data Availability

No new data were generated or analysed in support of this research.
