# Correction to: Interdisciplinary recommendations for recurrent hyperkalaemia: insights from the GUARDIAN-HK European Steering Committee

**DOI:** 10.1093/ehjcvp/pvag009

**Published:** 2026-02-04

This is a correction to: Gianluigi Savarese, María Jesús Izquierdo, Clara Bonanad, Aaron Wong, Roland Schmitt, Pietro Manuel Ferraro, Francesco Dentali, James O Burton, Giuseppe Rosano, Interdisciplinary recommendations for recurrent hyperkalaemia: insights from the GUARDIAN-HK European Steering Committee, *European Heart Journal - Cardiovascular Pharmacotherapy*, Volume 11, Issue 7, November 2025, Pages 630–637, https://doi.org/10.1093/ehjcvp/pvaf055

The following statement has been added to the **Funding** section: “This study was supported by the Italian Ministry of Health (Ricerca Corrente) 20/1819.”

