# Supplementary material for: Interdisciplinary recommendations for recurrent hyperkalaemia: insights from the GUARDIAN-HK European Steering Committee
Source: Eur Heart J Cardiovasc Pharmacother. 2025 Jul 21;11(7):630–7. doi: 10.1093/ehjcvp/pvaf055 (PMC12582656; doi:10.1093/ehjcvp/pvaf055)
Supplement: pvaf055_Supplementary_Data [file pvaf055_supplementary_data.docx]

# Supporting information

# Interdisciplinary recommendations for recurrent hyperkalaemia: Insights from the GUARDIAN-HK European Steering Committee

## Authors:

Gianluigi Savarese^1^; María Jesús Izquierdo^2^; Clara Bonanad^3^; Aaron Wong^4^; Roland Schmitt^5^; Pietro Manuel Ferraro^6^; Francesco Dentali^7^; James O. Burton^8^; Giuseppe Rosano^9^

## Affiliations:

^1^Department of Medicine, Karolinska Institute, Stockholm, Sweden; Heart and Vascular and Neurology Theme, Karolinska University Hospital, Stockholm, Sweden

^2^Department of Nephrology, Hospital Universitario de Burgos, Burgos, Spain

^3^Cardiology Department, Hospital Clínico Universitario de Valencia, Valencia, Spain

^4^Department of Cardiology, Princess of Wales Hospital, Bridgend, United Kingdom

^5^Department of Nephrology and Hypertension, University Hospital Schleswig-Holstein, Kiel, Germany

^6^Section of Nephrology, Department of Medicine, Università degli Studi di Verona, Verona, Italy

^7^Division of Internal Medicine, Medical Center, Ospedale di Circolo & Fondazione Macchi, ASST Sette Laghi, Varese, Italy

^8^Department of Cardiovascular Sciences, University of Leicester, Leicester, United Kingdom

^9^Department of Human Sciences and Promotion of Quality of Life, Chair of Pharmacology, San Raffaele University of Rome, Rome, Italy; IRCCS San Raffaele, Roma, Italy

## Corresponding author:

Gianluigi Savarese, Department of Medicine, Karolinska Institute, Stockholm, Sweden. [gianluigi.savarese@ki.se](mailto:gianluigi.savarese@ki.se)

## Supplementary Table S1: Guideline recommendations for the prevention of HK

| **Association** | **Reference** | **Year** | **Population** | **Preventing HK occurrence/recurrence** |
| --- | --- | --- | --- | --- |
| SIN/SIMI/FADOI | De Nicola et al. ^1^ | 2024 | On RAASi | Check serum potassium prior to initiating RAASi treatments, and then regularly throughout treatment.  Educate patients on HK, as well as the risks of discontinuing their current RAASi treatments and the need to avoid excessive dietary potassium intake.  A potassium intake of <3 g/day is recommended in patients with CKD and recurrent HK, while maintaining a healthy diet rich in fruits and vegetables.  A successful combination of optimal RAASi plus potassium binder can avoid/delay the need for dialysis and other serious clinical outcomes. |
| UK Kidney Association | Alfonzo et al. ^2^ | 2023 | Any | **Prevention of HK in the community:**  Consider low-potassium diet for patients with persistent serum potassium >5.5 mEq/L after non-dietary causes of HK (constipation, acidosis and poorly controlled diabetes) have been addressed.  Consider sodium bicarbonate in CKD patients with a serum bicarbonate level <22 mmol/L with or without HK, loop diuretics for the treatment of chronic HK in patients who are non-oliguric and volume replete, and exercise caution in prescribing trimethoprim to patients with renal impairment or those taking RAASi.  Consider patiromer or SZC (initiated in secondary care only) for persistent HK with a confirmed serum potassium ≥6.0 mEq/L in outpatients with CKD stage 3b–5 (not on dialysis) or HF receiving a suboptimal dose or not receiving RAASi therapy owing to HK.  Discontinue patiromer or SZC if RAASi therapy is stopped. Calcium resonium may be used as a short-term option to treat chronic HK in non-hospitalised patients who do not meet the criteria for patiromer or SZC.  Advise patients regarding the risks of AKI and HK during acute illness and measures to avoid these complications.  **Prevention of HK in hospitalised patients:**  Review medication that can cause HK in the context of the current illness and level of renal function both on and during hospital admission.  Consider low-potassium diet for hospitalised patients with moderate or severe HK after non-dietary causes of HK (constipation, acidosis and poorly controlled diabetes) have been addressed.  Evaluate the risk of recurrence of HK before reinstating previous medication that may have contributed to the episode. |
| KDIGO | KDIGO CKD Work Group^3^ | 2024 | CKD | In people with CKD and the management of non-emergent hyperkalaemia, a systematic approach of treating correctable factors (e.g. correction of severe metabolic acidosis) and understanding the role of diet and medications may provide a pragmatic framework.  For people with CKD and severe recurrent HK (potassium >6 mmol/L), balance the additional cost of potassium exchange agents against the health benefits and net savings of healthcare costs resulting from potassium-lowering treatment. The key is to implement a successful affordable strategy for HK management that allows the maintenance of other therapies.  Provide advice to limit the intake of foods rich in bioavailable potassium (e.g. processed foods) for people with CKD G3–G5 who have a history of HK or as a prevention strategy during disease periods in which HK risk may be a concern (the guideline does not endorse restrictions in early stages of CKD because high intake of foods naturally rich in potassium appears to be protective against disease progression, and dietary restriction of foods naturally containing potassium, such as fruits and vegetables, may be harmful to cardiac health) |
| KDIGO | KDIGO Diabetes Work Group^4^ | 2022 | CKD and diabetes | Moderate potassium intake, avoid potassium-containing salt substitutes or food products containing the salt substitutes.  Review medications and avoid drugs that can impair kidney excretion of potassium.  Avoid constipation (sufficient fluid intake and exercise).  Initiate diuretics treatment to enhance the excretion of potassium in the kidneys, especially when there is concomitant volume overload or hypertension.  Treat with oral sodium bicarbonate to minimise the risk of HK in patients with CKD and metabolic acidosis. Concurrent use of diuretics will reduce the risk of fluid overload that can occur from sodium bicarbonate treatment.  Consider treatment with potassium binders, such as patiromer or SZC, when other measures fail to control serum potassium levels. |
| KDIGO | KDIGO Blood Pressure Work Group^5^ | 2021 | Hypertension and CKD | Use caution in implementing **DASH-type diet or** potassium-containing salt substitutes in CKD populations, especially in those with advanced CKD, hyporeninaemic hypoaldosteronism or HK from other causes.  Consider dietary potassium restriction; discontinuation of potassium supplements, certain salt substitutes and drugs that cause HK; and adding potassium-wasting diuretics and oral potassium binders. |
| KDIGO | Lindner et al. ^6^ | 2020 | CKD | If the risk of HK remains an ongoing issue, provide dietary instructions regarding low-potassium foods and avoidance of potassium-rich foods to patients with moderate and severe kidney disease. |
| KDIGO | Clase et al. ^7^ | 2020 | CKD | Unable to provide evidence-based recommendations or suggestions on preferential strategies because of a lack of evidence for most of the strategies, the absence of evidence on comparative efficacy of alternative strategies and the potential for harm with at least some of them.  Approaches include dietary potassium restriction, permissive approach (no additions or changes to management despite awareness of HK), discontinuation of medications elevating potassium (e.g. RAASi), use of potassium-wasting diuretics, gastrointestinal potassium wasting and potassium binders. |
| ESH/ISH/ERA | Mancia et al. ^8^ | 2023 | Arterial hypertension | Do not combine ACEi, ARB, or aliskiren.  Consider a potassium binder in CKD patients with HK to maintain serum potassium levels <5.5 mEq/L and allow optimal treatment with a RAASi. |
| AHA/ACC/HFSA | Heidenreich et al. ^9^ | 2022 | HF | Use newer potassium binders to maintain serum potassium <5.5 mEq/L.  Avoid MRAs in patients with HFrEF and NYHA class II to IV symptoms if potassium is ≥5.0 mEq/L. |
| ESC | McDonagh et al. ^10^ | 2021 | HF | Potassium binders can be used for acute and chronic potassium lowering. In patients with chronic or recurrent HK on RAASi, an approved potassium-lowering agent may be initiated as soon as potassium levels are confirmed to be >5.0 mEq/L. Potassium-lowering treatment should be maintained unless alternative treatable aetiology for HK is identified.  Discontinue/reduce RAASi in patients with potassium levels of >6.5 mEq/L on either maximal or submaximal tolerated, guideline-recommended target dose of RAASi. |
| ESC | Rosano et al. ^11^ | 2018 | CVD treated with RAASi | Review diet, supplements and medications. Consider a low-potassium diet and loop or thiazide diuretics that increase potassium excretion. |
| JCS/JHFS | Kitai et al. ^12^ | 2025 | HF | Educate patients on a potassium-restricted diet  Reduce or discontinue medications that increase potassium levels of have nephrotoxic effects (e.g. non-steroidal anti-inflammatory drugs  Considered maintaining or initiating potassium binders to prevent recurrent HK, and administer, reintroduce, or uptitrate RAASi |

ACC, American College of Cardiology; ACEi, angiotensin-converting enzyme inhibitor; AHA, American Heart Association; AKI, acute kidney injury; ARB, angiotensin receptor blocker; CKD, chronic kidney disease; CVD, cardiovascular disease; DASH, **Dietary Approaches to Stop Hypertension;** ERA, European Renal Association; ESC, European Society of Cardiology; ESH, European Society of Hypertension; FADOI, Federation of Associations of Hospital Doctors on Internal Medicine; HF, heart failure; HFrEF, heart failure with reduced ejection fraction; HFSA, Heart Failure Society of America; HK, hyperkalaemia; ISH, International Society of Hypertension; JCS, Japanese Circulation Society; JHFS, Japanese Heart Failure Society; KDIGO, Kidney Disease: Improving Global Outcomes; MRA, mineralocorticoid receptor antagonist; NYHA, New York Heart Association; RAASi, renin–angiotensin–aldosterone system inhibitor; SIMI, Italian Society of Internal Medicine; SIN, Italian Society of Nephrology; SZC, sodium zirconium cyclosilicate.

## Supplementary Table S2: Summary of studies evaluating RAASi utilisation with potassium binders

| **Study /  Study type** | **Aim** | **Potassium binder** | **Study type** | **Number of participants** | **Follow-up** | **Effect of binder on RAASi utilisation** | **Effect of binder on potassium levels** |
| --- | --- | --- | --- | --- | --- | --- | --- |
| REALIZE-K, 2025^13^ | Evaluate the efficacy and safety of SZC in optimising spironolactone use patients with HFrEF and prevalent hyperkalaemia (or at high risk). | SZC | RCT | 102 treated with SZC  101 treated with placebo | 6 months total | More patients received spironolactone ≥25 mg/day on SZC (81%) than on placebo (50%; OR: 4.33; 95% CI: 2.50 to 7.52; P<0.001) (secondary endpoint)  Patients on SZC took longer to require a decrease or discontinuation of spironolactone due to hyperkalaemia than those on placebo (HR: 0.37; 95% CI: 0.17 to 0.73; P=0.006) (secondary endpoint) | Patients on SZC were more likely to maintain normokalaemia on spironolactone ≥25 mg/day without rescue therapy for hyperkalaemia compared with placebo (71% vs 36%; OR: 4.45; 95% CI: 2.89 to 6.86; P<0.001) (primary endpoint)  SZC (vs placebo) also improved the following secondary endpoints: normokalaemia on randomization dose of spironolactone and without rescue therapy (58% vs 23%; OR: 4.58; 95% CI: 2.78 to 7.55; P<0.001); time to HK (HR: 0.51; 95% CI: 0.37 to 0.71; P<0.001) |
| ZORA, 2023^14^ | Investigate RAASi utilisation after an episode of HK in patients with HF and/or CKD | SZC | RWD | 565 (USA), 776 (Japan), 56 (Spain) treated with SZC  2068 (USA), 2629 (Japan), 203 (Spain) without a potassium binder | 6 months total | Patients on SZC were more likely to maintain guideline-recommended RAASi therapy at 6 months following an HK episode compared with patients not treated with a potassium binder (OR: 2.56; 95% CI: 1.92 to 3.41; P<0.0001) |  |
| OPTIMIZE I, 2023^15^ | Evaluate RAASi modifications among patients who initiated SZC for HK | SZC | RWD | 589 | Mean follow-up = 244.5 days (SD: 98.9, median: 229.0) | Most patients (77.4%) optimised RAASi therapy after initiating SZC; 69.6% maintained the same dosage while 7.8% had up-titrations |  |
| PRIORITIZE-HF^a^, 2023^16^ | Evaluate the benefits and risks of using SZC to intensify RAASi therapy | SZC | RCT | 92 (SZC)  90 (placebo) | 12 weeks total | No statistically significant difference in the percentage of patients at RAASi target dose at 3 months between the groups (P=0.43) | Mean serum potassium concentrations during the treatment period were numerically lower in patients randomised to SZC than those in the placebo group |
| DIAMOND, 2022^17^ | Investigate the impact of patiromer on serum potassium level and its ability to enable specified target doses of RAASi use in patients with HF and reduced ejection fraction | Patiromer | RCT | 439 (patiromer)  439 (placebo) | Median follow-up was 27 (IQR: 13–43) weeks | Discontinuation or reduction of the target MRA dose occurred in 61 participants (13.9%) in the patiromer group and in 83 (18.9%) in the placebo group (HR: 0.62; 95% CI: 0.45 to 0.87; P=0.006) | Patients on patiromer had a lower total number of adjusted HK events/100 person-years (77.7 vs. 118.2 with placebo; HR: 0.66; 95% CI: 0.53 to 0.81; P<0.001) and lower potassium levels at study end, with a between-group difference of –0.10 mmol/L (95% CI: –0.13 to –0.07; P<0.001) |
| AMBER, 2019^18^ | Evaluate the use of patiromer to allow more persistent use of spironolactone in patients with CKD and resistant hypertension | Patiromer | RCT | 147 (patiromer)  148 (placebo) | 12 weeks total | More patients in the patiromer group were still receiving spironolactone at 12 weeks (86%) compared with the placebo group (66%) (P<0.001) |  |
| OPAL-HF, 2015^19^ | Evaluate the safety and efficacy of patiromer in patients with CKD who were receiving at least one RAASi and who had HK | Patiromer | RCT | 55 (patiromer)  52 (placebo) | 4 weeks run-in and 4 weeks randomised withdrawal phase (total) | More patients in the patiromer group were still receiving RAASi at the end of the randomised withdrawal phase (94%) compared with placebo-treated patients (44%) (no statistics provided) | The estimated median change in the potassium level from the start of the randomised withdrawal phase to week 4 of the phase was 0.72 mEq/L in the placebo group and 0 mEq/L in the patiromer group, with a between-group difference of 0.72 mmol/L (95% CI: 0.46 to 0.99; P<0.001)  More patients on placebo (91%) had at least one episode of potassium >5.0 mEq/L compared with those on patiromer (43%) (P<0.001) |
| PEARL-HF, 2011^20^ | Evaluate the efficacy and safety of patiromer on serum potassium levels in patients with HF receiving standard therapy and spironolactone | Patiromer | RCT | 56 (patiromer)  49 (placebo) | 28 days total | Significantly more patients in the patiromer group had their spironolactone dose increased compared with patients in the placebo group (91% vs. 74%; P= 0.019). | The patiromer group had significantly lowered serum potassium levels relative to the placebo group (difference between groups of −0.45 mEq/L; P<0.001)  Fewer patients in the patiromer group developed HK (serum potassium >5.5 mEq/L) compared with those on placebo (7% vs. 25%; P= 0.015) |

^a^The PRIORITIZE-HF study was terminated prematurely owing to the COVID-19 pandemic.

CI, confidence interval; CKD, chronic kidney disease; COVID-19, coronavirus disease 2019; HF, heart failure; HK, hyperkalaemia; HR, hazard ratio; IQR, interquartile range; MRA, mineralocorticoid receptor antagonist; OR, odds ratio; RAASi, renin–angiotensin–aldosterone system inhibitor; RCT, randomised controlled trial; RWD, real-word data; SD, standard deviation; SZC, sodium zirconium cyclosilicate.

## Supplementary Table S3: Causes of hyperkalaemia

|  | **Reversible** | **Non-reversible** |
| --- | --- | --- |
| **Reduced excretion of potassium** | Acute kidney injury (e.g. acute tubular necrosis or interstitial nephritis)  Reduced glomerular filtration rate owing to secondary causes (e.g. acute volume depletion from dehydration or bleeding)  Drugs that impair renin–angiotensin–aldosterone axis, such as renin–angiotensin–aldosterone inhibitors, or act through other mechanisms (e.g. trimethoprim)  Constipation | Chronic kidney disease (reduced glomerular filtration rate due to loss of normal kidney parenchyma [e.g. immune and non-immune-mediated kidney disorders])  Impaired renin–aldosterone axis (e.g. Addison's disease, aldosterone deficiency or aldosterone insensitivity)  Diabetes (hyporeninaemic hypoaldosteronism) |
| **Transcellular shift of potassium (intracellular to extracellular compartment)** | Cellular injury (e.g. rhabdomyolysis from crush injury or excessive exercise)  Metabolic acidosis (e.g. diabetic ketoacidosis or secondary to decreased tissue perfusion from sepsis or dehydration)  Tumours lysis syndrome  Drugs (e.g. succinylcholine) | Diabetes (insulin deficiency or resistance) |
| **Increased potassium intake** | High-potassium diet  Intravenous fluids (e.g. total parenteral nutrition)  Medications with high potassium content (e.g. potassium chloride, potassium iodide)  Massive blood transfusion | – |

## Supplementary Table S4: Tools for predicting HK

| **Population** | **Authors** | **Setting** | **HK definition** | **Prediction** | **Demographics** | **Laboratory** | **Comorbidities** | **Drugs** | **Other** |
| --- | --- | --- | --- | --- | --- | --- | --- | --- | --- |
| CKD starting lisinopril | Johnson et al 2010^21^ | Health maintenance organization | >5.5 mmol/L; or diagnosis code of HK | 90-days | Age | eGFR | Diabetes, HF | ACEi (starting dose), K supplements, K-sparing diuretics, ARBs | – |
| ACEi or ARB initiation | Bandak et al 2017^22^ | primary, secondary or tertiary care | >5.5 mmol/L | 1 year | Sex | eGFR, baseline K | Diabetes, HF | K-sparing diuretics | – |
| CKD | Sharma et al 2020^23^ | Population passed (insurance records) | >5.0 mmol/L; or diagnosis code of HK; or prescription for K binder | 1 year | Age, sex, geography | Baseline K, Hb | CKD stage, diabetes, HF, hyperlipidaemia, osteoporosis, PAD, malignancy, COPD | ACEi, MRA, Calcineurin inhibitor, RAASi dose, | – |
| Advanced CKD | Xue et al 2022^24^ | Tertiary centre | >5.5 mmol/L | 6 months | Age | Medical acidosis, previous HK | CKD stage, Diabetes, HF | RAASi | – |
| Advanced CKD | Chang et al 2023^25^ | Outpatients | >5.5 mmol/L | Next clinic (within 3 months) | – | Baseline K, BUN, Hgb, | HF | ARB, calcium polystyrene sulfonate | – |
| HD | Mei et al 2022^26^ | Tertiary | ≥ 5.5 mmol/L | – | – | Na, Ca, Phosphate, BUN | Diabetes | – | number of HD sessions |

ACEi, angiotensin converting enzyme inhibitor; ARB, aldosterone receptor blocker; BUN, blood urea nitrogen; COPD, chronic obstructive pulmonary disease; eGFR, estimated glomerular filtration rate; HD, haemodialysis; HF, heart failure; Hgb, haemoglobin; HK, hyperkalaemia; PAD, peripheral artery disease; RAASi, renin-angiotensin-aldosterone system inhibitor

## Supplementary Table S5: Recommendations regarding RAASi therapy in HK

| **Association** | **Reference** | **Year** | **Population** | **RAASi therapy in response to HK** |
| --- | --- | --- | --- | --- |
| SIN/SIMI/ FADOI | De Nicola et al. ^1^ | 2024 | On RAASi | Check serum potassium prior to initiating RAASi treatments, and then regularly throughout treatment.  Treat chronic HK first before reducing the dose or discontinuing RAASi treatment if serum potassium levels exceed 6.5 mmol/L.  Treatment should involve the use of newer potassium binders (i.e. SZC or patiromer), based on evidence that these agents can allow patients to effectively achieve normokalaemia while optimising RAASi treatment. |
| UK Kidney Association | Alfonzo et al. ^2^ | 2023 | Any | Assess urea and electrolytes prior to initiation of ACEi or ARB and use these drugs with caution if serum potassium is >5.0 mEq/L.  Avoid initiation of MRAs in patients with a baseline serum potassium >5.0 mmol/L or eGFR <30 mL/min.  Consider dose reduction of RAASi in patients with serum potassium between 5.5–5.9 mmol/L and cease RAASi in patients with serum potassium ≥6 mEq/L who do not meet the criteria for treatment with patiromer or SZC.  Withhold RAASi during acute illness (e.g. sepsis, hypovolaemia and/or AKI) at all severities of HK.  Minimise the duration of cessation of RAASi and communicate clearly after hospital discharge. Involve specialist services, and renal and heart failure teams, to facilitate safer re-introduction of treatment.  Consider patiromer or SZC (initiated in secondary care only) for persistent HK with serum potassium ≥6.0 mEq/L in outpatients with CKD stage 3b–5 (not on dialysis) or HF receiving a suboptimal dose or not receiving RAASi therapy owing to HK. |
| KDIGO | KDIGO CKD Work Group^3^ | 2024 | CKD | HK associated with use of ACEi or ARB can often be managed by measures to reduce the serum potassium levels rather than decreasing the dose or stopping ACEi or ARB.  Consider reducing the dose or discontinuing RAASi in the setting of uncontrolled HK despite medical treatment. Review and restart RAASi at a later date if patient condition allows.  **Avoid any combination of ACEi, ARB and direct renin inhibitor therapy in patients with CKD, with or without diabetes.** |
| KDIGO | KDIGO Diabetes Work Group^4^ | 2022 | CKD and diabetes | HK associated with the use of an ACEi or ARB can often be managed by measures to reduce serum potassium levels rather than decreasing the dose or stopping the ACEi or ARB immediately.  Reduce or discontinue ACEi or ARB therapy in uncontrolled HK despite medical treatment.  **Avoid any combination of ACEi, ARB and direct renin inhibitor therapy in patients with CKD, with or without diabetes.** |
| KDIGO | KDIGO Blood Pressure Work Group^5^ | 2021 | Hypertension and CKD | **HK associated with use of RAASi can often be managed by measures to reduce serum potassium levels rather than decreasing the dose or stopping RAASi.**  Consider reducing the dose or discontinuing ACEi or ARB in uncontrolled HK despite medical treatment.  MRAs should not be used in patients with high risk of HK (e.g. hypoaldosteronism or Type 4 renal tubular acidosis).  **Avoid any combination of ACEi, ARB and direct renin inhibitor therapy in patients with CKD, with or without diabetes.** |
| KDIGO | Lindner et al. ^6^ | 2020 | CKD | Consider temporarily discontinuing RAASi therapy.  Search for and treat any underlying condition leading to HK. |
| KDIGO | Clase et al. ^7^ | 2020 | CKD | Discontinue medications that elevate potassium levels (e.g. RAASi). |
| ESH/ISH/ ERA | Mancia et al. ^8^ | 2023 | Arterial hypertension | Avoid reducing the dose or discontinuing RAASi. |
| AHA/ACC/HFSA | Heidenreich et al. ^9^ | 2022 | HF | Discontinue MRA if potassium cannot be maintained <5.5 mEq/L.  In patients experiencing mild decrease of renal function during HF hospitalisation, GDMT should not routinely be discontinued.  In patients with HFrEF, if discontinuation of GDMT is necessary during hospitalisation, it should be reinitiated and further optimised as soon as possible. |
| ESC | McDonagh et al. ^10^ | 2021 | HF | The addition of an ARB (or renin inhibitor) to the combination of an ACEi and an MRA is not recommended in patients with HF, because of the increased risk of renal dysfunction and HK.  In those admitted with acute decompensated heart failure, oral OMT (class I recommended medical therapies for at least 3 months) should be continued, except if there is severely impaired renal function or HK, when a dose reduction or withdrawal should be considered. Optimise therapy before discharge.  In patients with chronic or recurrent HK not on maximal tolerated guideline-recommended target dose of RAASi, an approved potassium-lowering agent may be initiated as soon as confirmed potassium levels are >5.0 mEq/L. RAASi should be optimised when potassium levels are <5.0 mEq/L.  Discontinue/reduce RAASi in patients with potassium levels of >6.5 mEq/L on either maximal or submaximal tolerated, guideline-recommended target dose of RAASi. |
| ESC | Rosano et al. ^11^ | 2018 | CVD treated with RAASi | Titrate RAASi to maximum tolerated, guideline-recommended target dose.  Consider initiating an approved potassium-lowering agent as soon as potassium levels are >5.0 mEq/L.  Discontinue/reduce RAASi if potassium >6.5 mEq/L.  Carefully reintroduce RAASi as soon as possible while monitoring potassium levels. |
| JCS/JHFS | Kitai et al. ^12^ | 2025 | HF | If serum potassium is 5.0–5.4 mEq/L, consider potassium binders, especially when uptitrating RAASi  If serum potassium is 5.5–6.5 mEq/L, consider potassium binders, and reducing or discontinuing RAASi  If serum potassium is >6.5 mEq/L, initiate appropriate urgent treatment and consider reducing or discontinuing RAASi  Considered maintaining or initiating potassium binders to prevent recurrent HK, and administer, reintroduce, or uptitrate RAASi |

ACC, American College of Cardiology; ACEi, angiotensin-converting enzyme inhibitor; AHA, American Heart Association; AKI, acute kidney injury; ARB, angiotensin receptor blocker; CKD, chronic kidney disease; CVD, cardiovascular disease; eGFR, estimated glomerular filtration rate; ERA, European Renal Association; ESC, European Society of Cardiology; ESH, European Society of Hypertension; FADOI, Federation of Associations of Hospital Doctors on Internal Medicine; GDMT, guideline-directed medical therapy; HF, heart failure; HFrEF, heart failure with reduced ejection fraction; HFSA, Heart Failure Society of America; HK, hyperkalaemia; ISH, International Society of Hypertension; KDIGO, Kidney Disease: Improving Global Outcomes; MRA, mineralocorticoid receptor antagonist; OMT, optimal medical therapy; RAASi, renin–angiotensin–aldosterone system inhibitor; SIMI, Italian Society of Internal Medicine; SIN, Italian Society of Nephrology; SZC, sodium zirconium cyclosilicate.

## Supplementary Table S6: Potassium-monitoring recommendations

| **Association** | **Reference** | **Year** | **Population** | **Potassium monitoring** |
| --- | --- | --- | --- | --- |
| SIN/SIMI/FADOI | De Nicola et al. ^1^ | 2024 | On RAASi | Monitor serum potassium levels prior to initiating RAASi, and then regularly throughout treatment. |
| UK Kidney Association | Alfonzo et al. ^2^ | 2023 | Any | Patients known to have CKD, HF and/or diabetes, who are at risk of HK, should undergo regular blood monitoring at a frequency (2–4 times per year) dependent on level of renal function and degree of proteinuria.  Repeat serum potassium within 3 days, or as soon as feasible, if an episode of mild HK (potassium 5.5–5.9 mEq/L) is detected unexpectedly in the community.  Repeat serum potassium measurement within 1 day of an episode of moderate HK (potassium 6.0–6.4 mEq/L) when detected in the community.  Arrange community blood monitoring on discharge for all patients who have required treatment for HK during hospital admission.  Assess urea and electrolytes at prior to and 1–2 weeks after initiation of ACEi or ARB and after every dose titration.  Assess urea and electrolytes at 1 week after initiation of MRA or after dose up-titration, then monthly for the first 3 months, 3-monthly for the first year and 4-monthly thereafter.  Increase frequency of monitoring in patients treated with RAASi with a serum potassium between 5.5–5.9 mEq/L.  Consider weekly monitoring for the first month after every dose titration, and then monthly thereafter for patients treated with a potassium binder. |
| ACEP | Rafique et al. ^27^ | 2021 | Any | Close follow‐up after an episode of HK, ideally within 24–48 hours. |
| KDIGO | KDIGO CKD Work Group^3^ | 2024 | CKD | Monitor serum potassium within 2–4 weeks of starting or changing dose of a RAASi, and every 4 months after starting patients on non-steroidal MRA.  Repeat serum potassium levels within 24 hours in unexpected moderate HK (potassium levels 6.0–6.4 mEq/L) in adults who are clinically well or without acute kidney injury. |
| KDIGO | KDIGO Diabetes Work Group^4^ | 2022 | CKD and diabetes | **Monitor serum potassium within 2–4 weeks of initiation or increase in the dose of an ACEi or ARB.**  **Consider e**arlier monitoring (e.g. within 1 week) in patients at high risk of HK owing to low eGFR, history of HK, or borderline high serum potassium concentration.  Consider longer period for monitoring (e.g. after initiation but not dose titration) for patients at low risk of HK (e.g. patients with normal eGFR and serum potassium level). |
| KDIGO | KDIGO Blood pressure Work Group^5^ | 2021 | HTN and CKD | **Monitor serum potassium within 2–4 weeks of initiation or increase in the dose of a RAASi, depending on the current GFR and serum potassium.** |
| KDIGO | Lindner et al. ^6^ | 2020 | CKD | Timing of further monitoring of potassium concentration, and whether as an inpatient or as an outpatient, will depend upon the severity of the HK, the severity of its manifestations, the likelihood of recurrence and the patient’s overall clinical context and response to treatment. |
| KDIGO | Clase et al. ^7^ | 2020 | CKD | In patients at risk for HK, testing potassium concentration before and 1–2 weeks after initiation of RAASi therapy is recommended. |
| ESH/ISH/ERA | Mancia et al. ^8^ | 2023 | Arterial HTN | Closely monitor plasma potassium after RAASi treatment initiation and, depending on individual risk and the CKD stage, at least annually or at 3- to 6-month intervals thereafter.  Repeatedly monitor serum electrolytes within the first 4–8 weeks of treatment (depending on baseline kidney function) when initiating treatment. |
| AHA/ACC/HFSA | Heidenreich et al. ^9^ | 2022 | HF | Patients at risk for renal dysfunction or HK require close monitoring.  Regular checks of serum potassium levels and renal function should be performed according to clinical status, approximately 1 week, then 4 weeks, then every 6 months after initiating or intensifying MRA. |
| ESC | McDonagh et al. ^10^ | 2021 | HF | Follow up within 1 to 2 weeks after discharge for acute decompensated HF to monitor electrolytes.  Follow up at intervals no longer than 6 months to check electrolytes and renal function. For patients recently discharged from hospital, or in those undergoing up-titration of HF medication, follow-up intervals should be more frequent. |
| ESC | Rosano et al. ^11^ | 2018 | CVD treated with RAASi | Monitor patients on RAASi according to their clinical status.  Closely monitor serum potassium whenever potassium-lowering therapy is initiated for efficacy and risk of HK.  Maintain potassium-lowering treatment unless another aetiology for HK is identified. |
| JCS/JHFS | Kitai et al. ^12^ | 2025 | HF | Regularly monitor serum potassium and renal function |

ACC, American College of Cardiology; ACEi, angiotensin-converting enzyme inhibitor; ACEP, American College of Emergency Physicians; AHA, American Heart Association; ARB, angiotensin receptor blocker; CKD, chronic kidney disease; CVD, cardiovascular disease; eGFR, estimated glomerular filtration rate; ERA, European Renal Association; ESC, European Society of Cardiology; ESH, European Society of Hypertension; FADOI, Federation of Associations of Hospital Doctors on Internal Medicine; GFR, glomerular filtration rate; HF, heart failure; HFSA, Heart Failure Society of America; HK, hyperkalaemia; HTN, Hypertension; ISH, International Society of Hypertension; JCS, Japanese Circulation Society; JHFS, Japanese Heart Failure Society; KDIGO, Kidney Disease: Improving Global Outcomes; MRA, mineralocorticoid receptor antagonist; RAASi, renin–angiotensin–aldosterone system inhibitor; SIMI, Italian Society of Internal Medicine; SIN, Italian Society of Nephrology.

## References:

1. De Nicola L, Ferraro PM, Montagnani A, Pontremoli R, Dentali F, Sesti G. Recommendations for the management of hyperkalemia in patients receiving renin-angiotensin-aldosterone system inhibitors. *Intern Emerg Med* 2024;**19**:295–306. doi: https://doi.org/10.1007/s11739-023-03427-0

2. Alfonzo A, Harrison A, Baines R, Chu A, Mann S, MacRury M. *Clinical practice guidelines: Treatment of acute hyperkalaemia in adults*: UK Kidney Association; 2023.

3. Kidney Disease: Improving Global Outcomes CKD Work Group. KDIGO 2024 Clinical Practice Guideline for the Evaluation and Management of Chronic Kidney Disease. *Kidney Int* 2024;**105**:S117–S314. doi: https://doi.org/10.1016/j.kint.2023.10.018

4. Kidney Disease: Improving Global Outcomes Diabetes Work Group. KDIGO 2022 Clinical Practice Guideline for Diabetes Management in Chronic Kidney Disease. *Kidney Int* 2022;**102**:S1–s127. doi: https://doi.org/10.1016/j.kint.2022.06.008

5. Kidney Disease: Improving Global Outcomes Blood Pressure Work Group. KDIGO 2021 Clinical Practice Guideline for the Management of Blood Pressure in Chronic Kidney Disease. *Kidney Int* 2021;**99**:S1–s87. doi: https://doi.org/10.1016/j.kint.2020.11.003

6. Lindner G, Burdmann EA, Clase CM, Hemmelgarn BR, Herzog CA, Malyszko J, et al. Acute hyperkalemia in the emergency department: a summary from a Kidney Disease: Improving Global Outcomes conference. *Eur J Emerg Med* 2020;**27**:329–337. doi: https://doi.org/10.1097/MEJ.0000000000000691

7. Clase CM, Carrero JJ, Ellison DH, Grams ME, Hemmelgarn BR, Jardine MJ, et al. Potassium homeostasis and management of dyskalemia in kidney diseases: conclusions from a Kidney Disease: Improving Global Outcomes (KDIGO) Controversies Conference. *Kidney Int* 2020;**97**:42–61. doi: https://doi.org/10.1016/j.kint.2019.09.018

8. Mancia G, Kreutz R, Brunstrom M, Burnier M, Grassi G, Januszewicz A, et al. 2023 ESH Guidelines for the management of arterial hypertension The Task Force for the management of arterial hypertension of the European Society of Hypertension Endorsed by the International Society of Hypertension (ISH) and the European Renal Association (ERA). *J Hypertens* 2023. doi: https://doi.org/10.1097/HJH.0000000000003480

9. Heidenreich PA, Bozkurt B, Aguilar D, Allen LA, Byun JJ, Colvin MM, et al. 2022 AHA/ACC/HFSA Guideline for the Management of Heart Failure: A Report of the American College of Cardiology/American Heart Association Joint Committee on Clinical Practice Guidelines. *J Am Coll Cardiol* 2022;**79**:e263–e421. doi: https://doi.org/10.1016/j.jacc.2021.12.012

10. McDonagh TA, Metra M, Adamo M, Gardner RS, Baumbach A, Böhm M, et al. 2021 ESC Guidelines for the diagnosis and treatment of acute and chronic heart failure. *Eur Heart J* 2021;**42**:3599–3726. doi: https://doi.org/10.1093/eurheartj/ehab368

11. Rosano GMC, Tamargo J, Kjeldsen KP, Lainscak M, Agewall S, Anker SD, et al. Expert consensus document on the management of hyperkalaemia in patients with cardiovascular disease treated with renin angiotensin aldosterone system inhibitors: coordinated by the Working Group on Cardiovascular Pharmacotherapy of the European Society of Cardiology. *Eur Heart J Cardiovasc Pharmacother* 2018;**4**:180–188. doi: https://doi.org/10.1093/ehjcvp/pvy015

12. Kitai T, Kohsaka S, Kato T, Kato E, Sato K, Teramoto K, et al. JCS/JHFS 2025 Guideline on Diagnosis and Treatment of Heart Failure. *J Card Fail* 2025. doi: https://doi.org/10.1016/j.cardfail.2025.02.014

13. Kosiborod MN, Cherney DZI, Desai AS, Testani JM, Verma S, Chinnakondepalli K, et al. Sodium Zirconium Cyclosilicate for Management of Hyperkalemia During Spironolactone Optimization in Patients With Heart Failure. *J Am Coll Cardiol* 2025;**85**:971-984. doi: https://doi.org/10.1016/j.jacc.2024.11.014

14. Rastogi A, Pollack C, Sanchez-Lazaro IJ, Lesén E, Arnold M, Franzén S, et al. Contemporary Use of Guideline-Directed Medical Therapy and Associated Outcomes in Cardiorenal Patients with Hyperkalemia: An Observational Multi-Country Study [Poster presentation TH-PO358]. *ASN Kidney Week,* Philadelphia, PA., November 1–5, 2023.

15. Agiro A, An A, Cook EE, Mu F, Chen J, Desai P, et al. Real-World Modifications of Renin-Angiotensin-Aldosterone System Inhibitors in Patients with Hyperkalemia Initiating Sodium Zirconium Cyclosilicate Therapy: The OPTIMIZE I Study. *Adv Ther* 2023;**40**:2886–2901. doi: https://doi.org/10.1007/s12325-023-02518-w

16. Tardif JC, Rouleau J, Chertow GM, Al-Shurbaji A, Lisovskaja V, Gustavson S, et al. Potassium reduction with sodium zirconium cyclosilicate in patients with heart failure. *ESC Heart Fail* 2023;**10**:1066–1076. doi: https://doi.org/10.1002/ehf2.14268

17. Butler J, Anker SD, Lund LH, Coats AJS, Filippatos G, Siddiqi TJ, et al. Patiromer for the management of hyperkalemia in heart failure with reduced ejection fraction: the DIAMOND trial. *Eur Heart J* 2022;**43**:4362–4373. doi: https://doi.org/10.1093/eurheartj/ehac401

18. Agarwal R, Rossignol P, Romero A, Garza D, Mayo MR, Warren S, et al. Patiromer versus placebo to enable spironolactone use in patients with resistant hypertension and chronic kidney disease (AMBER): a phase 2, randomised, double-blind, placebo-controlled trial. *Lancet* 2019;**394**:1540–1550. doi: https://doi.org/10.1016/s0140-6736(19)32135-x

19. Weir MR, Bakris GL, Bushinsky DA, Mayo MR, Garza D, Stasiv Y, et al. Patiromer in patients with kidney disease and hyperkalemia receiving RAAS inhibitors. *N Engl J Med* 2015;**372**:211–221. doi: https://doi.org/10.1056/NEJMoa1410853

20. Pitt B, Anker SD, Bushinsky DA, Kitzman DW, Zannad F, Huang IZ, et al. Evaluation of the efficacy and safety of RLY5016, a polymeric potassium binder, in a double-blind, placebo-controlled study in patients with chronic heart failure (the PEARL-HF) trial. *Eur Heart J* 2011;**32**:820–828. doi: https://doi.org/10.1093/eurheartj/ehq502

21. Johnson ES, Weinstein JR, Thorp ML, Platt RW, Petrik AF, Yang X, et al. Predicting the risk of hyperkalemia in patients with chronic kidney disease starting lisinopril. *Pharmacoepidemiol Drug Saf* 2010;**19**:266–272. doi: https://doi.org/10.1002/pds.1923

22. Bandak G, Sang Y, Gasparini A, Chang AR, Ballew SH, Evans M, et al. Hyperkalemia After Initiating Renin-Angiotensin System Blockade: The Stockholm Creatinine Measurements (SCREAM) Project. *J Am Heart Assoc* 2017;**6**:e005428. doi: https://doi.org/10.1161/JAHA.116.005428

23. Sharma A, Alvarez PJ, Woods SD, Dai D. A Model to Predict Risk of Hyperkalemia in Patients with Chronic Kidney Disease Using a Large Administrative Claims Database. *Clinicoecon Outcomes Res* 2020;**12**:657–667. doi: https://doi.org/10.2147/ceor.S267063

24. Xue C, Zhou C, Yang B, Ye X, Xu J, Lu Y, et al. A Nomogram to Identify Hyperkalemia Risk in Patients with Advanced CKD. *Kidney360* 2022;**3**:1699–1709. doi: https://doi.org/10.34067/kid.0004752022

25. Chang HH, Chiang JH, Tsai CC, Chiu PF. Predicting hyperkalemia in patients with advanced chronic kidney disease using the XGBoost model. *BMC Nephrol* 2023;**24**:169. doi: https://doi.org/10.1186/s12882-023-03227-w

26. Mei Z, Chen J, Chen P, Luo S, Jin L, Zhou L. A nomogram to predict hyperkalemia in patients with hemodialysis: a retrospective cohort study. *BMC Nephrol* 2022;**23**:351. doi: https://doi.org/10.1186/s12882-022-02976-4

27. Rafique Z, Peacock F, Armstead T, Bischof JJ, Hudson J, Weir MR, et al. Hyperkalemia management in the emergency department: An expert panel consensus. *J Am Coll Emerg Physicians Open* 2021;**2**:e12572. doi: https://doi.org/10.1002/emp2.12572
